# Magnetically Targeted Stem Cell Delivery for Regenerative Medicine

**DOI:** 10.3390/jfb6030526

**Published:** 2015-06-30

**Authors:** Jhon Cores, Thomas G. Caranasos, Ke Cheng

**Affiliations:** 1 Joint Department of Biomedical Engineering, UNC-Chapel Hill & NC State University, NC 27606, USA; E-Mail: CoresJR@gmail.com; 2 Department of Molecular Biomedical Sciences, College of Veterinary Medicine, North Carolina State University, Raleigh, NC 27607, USA; 3 Division of Cardiothoracic Surgery, University of North Carolina at Chapel Hill, Chapel Hill, NC 27599, USA; E-Mail: thomas_caranasos@med.unc.edu

**Keywords:** SPION, magnetic targeting, stem cell, magnetic nanoparticle, regenerative medicine

## Abstract

Stem cells play a special role in the body as agents of self-renewal and auto-reparation for tissues and organs. Stem cell therapies represent a promising alternative strategy to regenerate damaged tissue when natural repairing and conventional pharmacological intervention fail to do so. A fundamental impediment for the evolution of stem cell therapies has been the difficulty of effectively targeting administered stem cells to the disease foci. Biocompatible magnetically responsive nanoparticles are being utilized for the targeted delivery of stem cells in order to enhance their retention in the desired treatment site. This noninvasive treatment-localization strategy has shown promising results and has the potential to mitigate the problem of poor long-term stem cell engraftment in a number of organ systems post-delivery. In addition, these same nanoparticles can be used to track and monitor the cells *in vivo*, using magnetic resonance imaging. In the present review we underline the principles of magnetic targeting for stem cell delivery, with a look at the logic behind magnetic nanoparticle systems, their manufacturing and design variants, and their applications in various pathological models.

## 1. Introduction

Stem cells have been studied for their ability to replicate undifferentiated daughter cells that can be physiologically induced to mature under specific conditions. Characterized by multi or pluripotency, they have the ability to differentiate into a plethora of specialized cells and confer therapeutic benefits that have catalyzed the field of regenerative medicine [1,2]. They have been shown to regenerate cardiomyocytes in myocardial infarction models, reendothelialize stented blood vessels, and induce vasculogenesis [3,4,5]. They can be used to treat retinal degenerations, spinal cord injuries, and are even being studied for their potential to treat brain traumas [6,7,8]. In adults, somatic stem cells localized within organs such as the brain and muscles help to regenerate tissues that have been damaged from wear and tear, diseases, and injuries [1,2]. With the advancements in cell-based therapies at the helm of biomedical endeavors, the shift in paradigm from one of transplantable organs to one of healable or even creatable organs is a foreseeable, albeit still elusive possibility that holds much promise for the 78,000 patients actively awaiting an organ transplant [9].

One of the most pressing problems with stem cell treatments, however, is the delocalization of the stem cells from the injury site over time. Inefficient engraftment can result from difficulties with homing to the injury site when administered intravenously, when the administered dosage does not comply with recommendations for patient specifications, or as a result of the innate mechanical stresses present at the target site [10]. 

Table 1 summarizes the imaging modalities and contrast agents that can be used to track cellular migration and lineage *in vivo*. The many imaging technologies available for research translates into and facilitates a burgeoning interest in the development of biocompatible tracers or contrast agents. Ongoing research into superparamagnetic iron oxide nanoparticles (SPIONs) used in conjunction with magnetic resonance imaging (MRI) has demonstrated that it is possible to combine noninvasive magnetic manipulation and imaging tracking in real time and *in vivo* [11]. Magnetic nanoparticles are well documented for their applications in detecting, diagnosing, and treating disease [7,10,12,13,14,15,16]. In addition, MRI is particularly favorable for clinical applications because unlike CT, PET, or X-Ray techniques, it does not use radiation or radioactive dyes, permitting longitudinal data acquisition on the same patient. Moreover, its ability to image soft and hard tissues enhances its utility in imaging cells in different physiological niches, and the high resolution of the data allows for the quantification of cells [17].

In this review, we will discuss the basic principles of magnetic targeting and elaborate on the components in the system of magnetically-targeted stem cell delivery, namely the magnetic nanoparticle itself, the magnetic field gradients and their function, synthesis and stabilization methods, applications in organ damage therapeutics, and the technology’s potential as a launching point for the evolution of future magnetic targeting techniques.

**Table 1:** Advantages and disadvantages of different imaging techniques and their contrast agents used for *in vivo* cellular tracking.

Imaging Modality	Contrast Agent	Advantages	Disadvantages	Ref.
MRI	Superparamagnetic iron oxide nanoparticles (SPIONs)	Painless, full body 3D scanning, no ionizing radiation is used, difficult but possible quantification of cells, manipulation of cells using external magnetic field	Tracer dilutes when cell divides, possible transfer of agent to other cells, not usable in patients with implants, imaging process can be claustrophobic	[17,18]
MRI	Gadolinium, fluorescent agents, and perfluorocarbon	Full body 3D scanning, no ionizing radiation, detection of individual cell is possible	Tracer dilutes when cell divides, possible transfer of agent to other cells, not usable in patients with implants, imaging process can be claustrophobic	[17,18]
Optical	Protein fluorescent markers, fluorescent dyes, luciferase substrates, and near infrared fluorophores	Extended palette of fluorophores permits simultaneous analysis of different cell types and lineages, can be combined with other imaging modalities, does not use ionizing radiation	Dye cytotoxicity, tracer dilutes when cell divides, tissue penetration depth is limited	[17,19,20]
PET & SPECT	SPECT: High-energy gamma emitters; PET: High-energy positron emitters	High detail, full body 3D scanning, transgenic approaches translate to no cell division dilutions in tracer signal, quantification is possible with SPECT	Ionizing radiation, quantification can be difficult in PET, genetic modification of stem cells, intravenous injection of contrast agent, radioactive tracer can cause allergic reaction	[17,21]
Ultra-Sound	Microbubbles	No ionizing radiation, possible to detect single cells, fast, relatively inexpensive, can image soft tissues	Low resolution, restricted to specific parts of the body, quantification is difficult, contrast agent dilutes when cell divides and can transfer to other cells	[17,22]
X-ray & CT	High density iodine or gadolinium	X-ray is fast and relatively inexpensive, CT permits full body, 3D scanning	High contrast agent concentrations can be toxic, ionizing radiation in dyes, X-ray data is hard to quantify, increase in possibility of cancer development at later age	[17,23]

## 2. Magnetic Targeting: Basic Principles, Components, Production, and Coatings

Magnetic stem cell targeting relies on the external or internal application of a magnetic field and its influence on a magnetic reactive carrier. A magnetically reactive particle is one in which its atoms can align the atomic magnetic moments parallel to the direction of the applied magnetic field [13]. Superparamagnetism, or a particle’s potential to strongly magnetize upon exposure to a magnetic field with negligible remanence is a highly desirable trait, enabling prevention of particle aggregation after the targeting is achieved. Magnetic remanence, or the amount of residual magnetism left over after a magnetic field is applied and subsequently removed, is imperative for the proper activation and deactivation of magnetic functionality of the nanoparticle. While the magnetization potential of superparamagnets is not as strong as that of ferromagnets, at room temperature, superparamagnetic nanoparticles exhibit relatively little particle aggregation, allowing for greater control during handling and administration [13,14,24]. 

The force of attraction between the magnetic nanoparticles and the source of magnetic field gradient is governed by the dot product of $\vec{m}$ and $\nabla \vec{B}$ as per the following equation [13,25]:

(1) $${\vec{F}}_{mag}=\left(\vec{m}\cdot \nabla \vec{B}\right)\vec{B}$$

where ${\vec{F}}_{mag}$ is the force generated on a magnetic carrier with a magnetic moment $\vec{m}$, $\vec{B}$ is the total magnetic field, and $\nabla \vec{B}$ are high field magnetic gradients.

### 2.1. Magnetic Nanoparticle

Superparamagnetic iron oxide nanoparticles have previously been studied for biomedical applications (e.g., drug delivery, medical imaging, and regenerative medicine) [15,26,27,28]. They are typically composed of a magnetite (Fe_3_O_4_) or maghemite (γ-Fe_2_O_3_) core. Both are naturally ferromagnetic in bulk, meaning they are permanently attracted to magnets or are permanently magnetic, but at diameters smaller than their intrinsic superparamagnetic radius and greater than their single domain radius, they become superparamagnets [24,29]. Classification of these superparamagnets depends on the field of study as well as their application. In the field of medicine and biology, they are categorized broadly by size; (50–180 nm) superparamagnetic iron oxide NPs (SPIONs), (10–50 nm) ultra-small superparamagnetic iron oxide NPs (USPIONs), and (<10 nm) very small superparamagnetic iron oxide NPs (VSPIONs) [29,30].

Magnetic nanoparticles have played an important role in MR imaging. In fact, the precedent for their implementation in regenerative medicine stems from their use as contrast agents in MRI [14], an imaging modality aptly suited for soft tissue imaging. Up until the adaptation of SPIONS as contrast agents in MRI, the relatively low sensitivity of the standard gadolinium chelate contrast agent made MR imaging unsuitable for molecular imaging. Gadolinium chelates only have an imaging sensitivity in the micromolar range, while SPIONs are sensitive in the nanomolar range and can be detected by T1, T2, and T2* MRI parameters vividly, due in part to their favorable relaxivity properties [31].

SPIONs have negligible side effects [32]. Iron containing nanoparticles show an acceptable level of biocompatibility in part due to the body’s innate ability to metabolize naturally occurring iron in the form of ferritin, and because they are superparamagnetic, the risk of particle agglomeration and thus vessel occlusion, is minimized. Further biocompatibility can be achieved by coating the cores with both inorganic and organic polymers.

As of 2012, the only FDA approved and clinically viable SPIONs in the US market are Gastromark and Ferumoxytol [29]. Gastromark (50 nm), also known as Lumirem, was approved by the FDA in 1996 as an orally administered MRI contrast agent. Ferumoxytol (20–50 nm), also known as Feraheme, was approved by the FDA in 2009 as an intravenously administered nanoparticle for the treatment of iron deficiency in adults with kidney disease. Of the two, Feraheme has attracted the most interest for studies on the magnetically targeted delivery of stem cells. In 1996, the FDA also approved the use of Feridex, another contrast agent for MR imaging of liver lesions but it was subsequently removed from the market for commercial use in 2009 due to lack of sales [33]. There are myriad other non-clinical SPIONs being researched both *in vitro* and *in vivo* for laboratory use, but none other has gained FDA approval for clinical trials. For an extensive tabulation of commercial and noncommercial SPIONs used in cellular and molecular imaging, see [32].

### 2.2. Synthesis of Magnetic Nanoparticles

There are a number of ways in which magnetic nanoparticles can be synthesized. Each offers discerning benefits in terms of quality, size distribution, and stability of the particles formed. Below is a brief description of the most common processes reported in the literature with a look at their advantages and drawbacks.

#### 2.2.1. Co-Precipitation

The synthesis of iron oxides using co-precipitation works by reacting aqueous salt solutions of either Fe^2+^ or Fe^3+^ with a base at room temperature in an inert atmosphere to form magnetite (Fe_3_O_4_), which is then easily oxidized into maghemite γ-Fe_2_O_3_, its stable counterpart [15,34]. Nanoparticle properties, including shape, size, and composition depend on the type of salt used in the reaction. Thus, iron chlorides, sulfates, and nitrates will confer different qualities onto the iron oxide particles formed. The co-precipitation process is relatively simple, which makes it among the most popular methods for producing iron oxide nanoparticles [35]. Its main drawback is its inability to produce a narrow particle size distribution. While it is possible to control the size of the particle by altering stirring speeds and synthesis temperatures during production, the size distribution will vary within the range of one order of magnitude.

Superparamagnetism depends on a magnetic property called blocking temperature, which is a factor of particles size, as well as the effective anisotropy constant, the applied magnetic field, and the experimental measuring time [15,32]. Above this temperature, the thermal energy of the particles is high enough to randomize their magnetic moments, leading to a superparamagnetic state; below this temperature, they behave like permanent magnets. The magnetic blocking temperature formula is derived from the Neel-Arrhenius relaxation time equation [15]:

(2) $$\tau =\tau_{0}e^{\frac{K_{eff}V}{k_{B}T}}$$

(3) $$T_{B}=\frac{K_{eff}V}{k_{B}\text{ln}\left(\frac{\tau_{m}}{\tau_{0}}\right)}$$

In this equation, τ, or relaxation time, is the mean amount of time it takes for a single-domain magnetic nanoparticle to flip its magnetic moment, τ_0_ is a constant attempt time with a value between 10^−9^ and 10^−10^ seconds, *K_eff_* is the magnetic anisotropy energy density, *T* is the measurement temperature, *V* is the volume of the particle, and *k*_B_ is the Boltzmann constant. In the blocking temperature equation, relaxation time is treated as a fixable variable indicating measurement time τ_m_. Body temperature and the Boltzmann constant are also fixed quantities, thus the size of the particles and the magnetic field applied on them will be the most relevant variables in determining a nanoparticle’s superparamagnetism. The fact that particle size varies greatly with co-precipitation translates to particles with different superparamagnetic thresholds [15]. Fortunately, iron’s low magnetocrystalline anisotropy means co-precipitation can still be used to produce superparamagnetic nanoparticles despite their polydispersion [35].

#### 2.2.2. Thermal Decomposition

Thermal decomposition occurs when a substance breaks down into its component parts under the influence of heat [15,34]. Metals are decomposed in boiling organic solvents that contain stabilizing surfactants. Such metallic precursors include metal cupferronates, carbonyls, and acetylacetonates. Typical surfactants include fatty acids, hexadecylamine, and oleic acid. While thermal decomposition is energy consuming, the ability to manipulate reagents affords size and morphology control in the resultant iron oxide nanoparticle, making it a valuable technique. However, like those produced by co-precipitation, these nanoparticles are also easily oxidized in air, a property that adds diametric length and requires consideration when gaging particle size. 

#### 2.2.3. Microemulsion

Microemulsions are dispersions of two immiscible liquids in which surfactants are used to stabilize one or both [34]. For instance, this technique can be used to produce iron oxide nanoparticles in reverse micelles of cetyltrimethylammonium bromide (surfactant), 1-butanol (cosurfactant), and octane (oil phase). While the resultant particles can be as small as 2 to 5 nm in diameter, the sizes can range greatly and the yield is low compared to co-precipitation and thermal decomposition methods.

#### 2.2.4. Hydrothermal Synthesis

The hydrothermal synthesis method of nanoparticle production depends on a liquid-solid-solution reaction. The system, reported by Li *et al.*, reacts metal linoleate, ethanol linoleic acid, and water-ethanol solution [34]. The reaction is done under hydrothermal conditions at varying temperatures and relies on an interfacial phase transfer and separation mechanism at the liquid-solid, and solid-solution interphases. With this method, Fe_3_O_4_ nanoparticles in the range of 9 to 12 nm can be produced with size uniformity and very good shape control. 

### 2.3. Magnetic Field Gradient

Along with a magnetically responsive SPION, the magnetic targeting system requires a magnetizing magnetic field and field gradient. The role of the magnetic field gradient is to attract and position stem cell/SPION complexes within the domain of the targeted pathological site. The magnetic targeting field and field gradient are most frequently applied using an externally placed magnet, but this set-up has inherent limitations. The magnetic flux density is greatest at the magnetic pole face and dissipates as you move farther away from the magnet [36]. Thus, if your targeted treatment focus is beyond the reach of the magnetic field (deep tissue), the SPION-loaded cells will not be attracted. This limitation has prompted research developing magnetizable implants in conjunction with the application of a homogeneous magnetic field for generation of deep tissue high field gradients around the implant [25]. While placing magnets within tissue does achieve a greater magnetic attraction, deeper tissue penetration is not feasible for every tissue type or organ such as the heart, kidney, and liver [37]. 

In order to overcome the limitation of magnetic field reach in organs where deep tissue placement of magnets is unfeasible, Huang *et al.* developed a magnetic system that focuses the magnetic field density at a distance from the magnet [36,37]. Their experiment used a small diameter tube to mimic a blood vessel, through which they injected magnetically labeled mesenchymal stem cells (MSCs) at different velocities. Their magnetic device would capture the cells as they flowed through the tube in a magnetic pocket focused 5 mm from magnetic field origins. The success of the device in capturing SPION-labeled cells varied positively with the magnetic flux density and negatively with the velocity of flow. 

Another feature inherent to permanent external magnetic fields is their non-uniformity along their tangential axes. Take for example the cylindrical magnet used by Huang *et al.* Along the vertical access, the strength of the field decreases with distance from the magnet as has been established, but along the horizontal axis the field polarizes at local radii [38]. In other words, the magnetic field creates circular rings of attraction at specific distances away from the magnet’s center. These uniformity and dissipation phenomena must be manipulated in order to achieve a viable clinical treatment using SPION-labeled cells. 

### 2.4. Protection and Stabilization of Nanoparticles

Despite the successful production of quality nanoscale particles for both bench-top and bedside applications, there are properties that make nano-sized iron particles difficult to work with. For instance, their innate instability in air causes them to oxidize, a process that alters the size of the particle. They can agglomerate, resulting in elimination from the body by the reticuloendothelial system [12,14,29,34,39]. In addition, because iron is a physiologically present element, the body is equipped with the mechanisms necessary for its metabolic digestion, which, depending on the length of the desired study or treatment, can dwindle the iron present at the target site. Moreover, high iron ion concentrations, with the potential to cross both nuclear and mitochondrial membranes, can be locally toxic to the body, particularly in individuals with genetic predispositions [40]. Free iron in the mitochondria can react with hydrogen peroxide and oxygen to produce hydroxyl radicals and ferric ions, which are highly reactive. Hydroxyl radicals, for instance, have been shown to damage DNA, proteins, polysaccharides, and lipids [30]. Thus, researchers apply protective coatings to iron oxide nanoparticles in search of favorable properties, including increased biocompatibility [39]. While much research has been done on a number of coatings (mild oxidation, surfactant, precious metal, silica, carbon coatings, cellulose, chitosan, and others) [32], the bulk of the literature points to three prominent SPION coating types: polyethylene glycol (PEG), starch, and dextran [41]. For an extensive list of SPION coatings, see [32].

#### 2.4.1. Polyethylene Glycol

Polyethylene Glycol is an amphiphilic polymer that anchors onto iron oxide through covalent bonds [34]. Once merged, the resultant nanoparticles are less susceptible to non-specific macrophage uptake, exhibit negligible aggregation, are more biocompatible, and resist corrosion in air and in hydrophilic environments [39].

#### 2.4.2. Dextran

Dextran is a naturally occurring glucose polymer with a repeating linear, alpha linked glucopyranose backbone. It has long been used in the preparation of iron therapies for human and animal anemias. The coupling of the dextran coating to the iron oxide nanoparticle core is done via non-covalent bonding, which can lead to de-shelling under some physiological conditions [28]. In order to ensure proper bonding between the iron oxide core and dextran shell, the dextran is carboxylated [28,42]. These nanoparticles have been studied extensively, particularly for their use in MRI. They have the ability to undergo surface modifications (antibody, peptide, small molecule derivatives), have favorable circulating half-lives, and are biocompatible.

#### 2.4.3. Starch

Starch is a hydrophilic polymer of D-glucose and the most common polysaccharide in nature. It is made up of a long chain alpha-linked polymer backbone of amylose. Amylopectin is its branched form. Starch-coated iron oxide nanoparticles are made through co-precipitation of Fe^2+^ and Fe^3+^ in starch solution [41]. In addition to starch and iron oxide, sodium hydroxide is added to the reaction in order to improve the coating coverage and further reduce agglomeration of the particles with one another. The three dimensional polymeric matrix that forms around the iron oxide core prevents oxidation, facilitates absorption into cells, is biodegradable, biocompatible, and non-toxic [32,41].

#### 2.4.4. Citrate

Efficient labeling of stem cells without transfection agents can be problematic. This is especially true for cell types that lack substantial phagocytic capacity, such as MSCs. As a result, poor intracellular SPION uptake and limited MRI sensitivity is achieved in these cells. In order to enhance cellular uptake, high concentrations of SPIONS or potentially toxic transfection agents are required to achieve magnetic labeling in MSCs and other non-phagocytic cells. Citrate, a relatively new type of polymer coating, can be used as an anionic monomer to improve cellular uptake and enhance MRI imaging [43]. The high negative charge of the citrate surface modification creates a strong affinity between the SPION and the cell, which translates to better internalization at lower concentrations without the need for transfection agents that can be cytotoxic if the ratio of transfection agent to SPIONS is not carefully titrated. Citrate has been shown to affect chondrogenic differentiation and chemotaxis at dose-dependent levels. Thus, further dosage studies must be conducted to preserve stem cell properties and before commercialization of citrate is possible.

## 3. Tagging Stem Cells with Iron Oxide Nanoparticles

**Figure 1:**
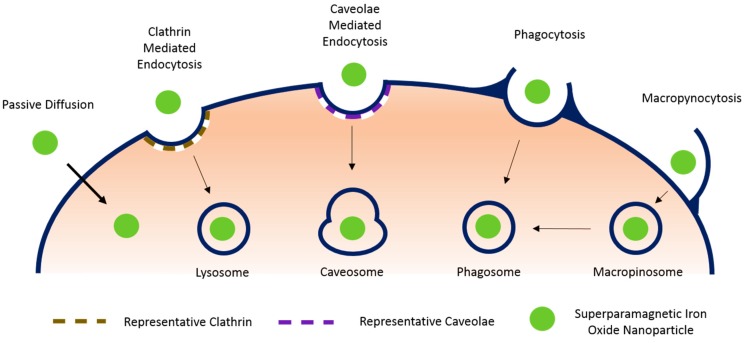
Representation of available mechanisms for SPION uptake by stem cells.

The internalization of SPIONs in stem cells can be accomplished in a number of ways. The technique used can determine the affordability, scale-up potential, and efficacy of the magnetic stem cell system [12]. Particle size, shape, and surface modifications will influence the rate at which a particle is internalized, the method used by the cell, and the area of the cell in which that particle ends up [29,44]. There are also cellular phenotypic considerations, as some cells will respond to different stimuli and not all are equipped with the tools to internalize a particle in the same way. Manipulations of surface charges using antibodies, peptides, and aptamers or via coatings can speed up the internalization [29]. Illustrated in Figure 1 and summarized below are the main cellular internalization methods available to a stem cell for the uptake of SPIONs.

### 3.1. Passive Diffusion

This energy independent internalization mechanism is achieved through electrochemical or concentration gradients. It is used by cells for small (20 nm) positively charged nanoparticles [45]. In an effort to stimulate and utilize passive diffusion for nanoparticle internalization, researchers are studying the manipulation of surface modifications with the potential to improve their uptake by cells. In a study intended to mimic cell-penetrating peptides, which can be internalized by energy-free mechanisms, Martin *et al.* and Calvin *et al.* demonstrated an improvement in cellular uptake of nanoparticles via guanidine dendritic structure and synthetic peptides [29]. 

### 3.2. Endocytosis

This is the most common way to load SPIONs into the cells. Generally, cells will be co-incubated with SPIONs for 12–48 h [11,38,46]. Cellular uptake of the SPIONs can be verified by methods such as Prussian blue staining [24,27,37,47]. In some instances, internalization can be facilitated by physicochemical methods such as electroporation [24,33] and/or in the presence of transfection reagents such as poly-L-lysine [29,37]. Endocytosis occurs in virtually all cells. The four main mechanisms by which endocytosis takes place are clathrin mediated, caveolae mediated, macropynocytosis, and a host of other clathrin and caveolae independent variants [44]. Phagocytosis is one such variant that occurs only in specific types of cells. The following summarizes the most common form of endocytosis (clathrin mediated), and three common variants. 

#### 3.2.1. Clathrin Mediated Endocytosis

The protein clathrin plays a principal role in the formation of coated vesicles and serves as the main mechanism for the internalization of macromolecules in most cell types [44]. Endocytosis mediated by clathrin is either receptor dependent or independent. Irrespective of which variant a cell uses, the general mechanism is the same. Upon nanoparticle interaction with the cellular membrane, the three heavy chains [48] that make up the clathrin structure join to form a polygonal lattice that deforms the membrane. A clathrin lattice develops around the nanoparticle until a vesicle is formed. That vesicle breaks down, eventually leaving the nanoparticle engulfed in an acidic and enzyme-rich cytoplasmic lysosome.

#### 3.2.2. Caveolae Mediated Endocytosis

Caveolae are flask-shaped membrane invaginations lined by caveolin, a dimeric protein. They are abundant in certain cell types, such as endothelial cells, smooth muscle cells, and fibroblasts. Cells use caveolae mediated endocytosis as an alternate pathway to internalizing nanoparticles in a regulated signaling process. During caveolae mediated endocytosis, caveolae form a vesicle that engulfs the nanoparticle, but instead of eventually forming a lysosome, the caveolar mediated pathway forms a caveosome, which is not rich in degrading enzymes [44]. 

#### 3.2.3. Phagocytosis

Occurring primarily in specialized cells like macrophages and monocytes, phagocytosis is a specific type of endocytosis that can be broken up into three distinct stages. The foreign nanoparticle is first tagged by proteins called opsonins, which make them visible to the cell. The opsonized particles then attach to the cell surface via receptor-ligand interactions. A series of signaling cascades initiate after receptor ligation and cellular extensions begin to engulf the particle into the cytoplasm in the phagosome that forms. The rate of this process is a factor of the surface properties of the particle, as well as its size, shape, and the way in which it interacts with opsonins and the cell membrane [44]. 

#### 3.2.4. Macropynocytosis 

Macropynocytosis generates large vesicles called macropinosomes through a clathrin independent pathway that does not appear to exhibit any selectivity. It occurs in many cell types. During macropynocytosis, actin driven membrane protrusions collapse onto the plasma membrane and fuse with it to internalize large particles [44]. 

### 3.3. External Binding (Antibody Mediated Targeting)

Chen *et al.* have developed an alternative method that obviates the need for cellular internalization of SPIONs prior to application at an injury site in New Zealand rabbits [12]. The group coated magnetic nanoparticles (MNP) with polyethylene glycol and used the active carboxy groups of the PEG surface to bind anti-CD34 antibodies; CD34 is a hematopoietic stem cell surface marker. In addition to binding the antibodies, the hydrophilic polymer PEG attenuates the agglomeration of the nanoparticles to one another, and minimizes protein adsorption onto its surface. The PEG coated, anti-CD34 treated MNPs were then injected into the blood stream of the rabbits where they bound to CD34^+^ stem cells. Once bound to the MNPs, the stem cells were guided to the injury site by applying a magnetic field gradient [12]. The advantage of using stem cell specific MNPs is that they obviate the need to isolate and expand cells in culture before tagging *in vitro*, an expensive and time-consuming procedure that is not viable in emergency situations. 

## 4. Application of SPIONs in Pathological Models

Application of SPIONs in pathological models is summarized in Table 2.

**Table 2:** Summary of the literature review on the application of SPION-labelled stem cells for the treatment of a plethora of pathological conditions.

Cell Type	Organ	Condition	Model	SPION Type	Ref.
Mesenchymal stem cell	Spine	Spinal cord injury	Unspecified rat	poly-L-lysine-coated SPIONs	[7]
		Spinal cord injury	Sprague–dawley rat	poly-L-lysine-coated SPIONs	[49]
	Heart	Myocardial infarction	Sprague–dawley rat	SPIO plus poly-L-lysine	[47]
	Vasculature	Coronary embolization	Sprague–dawley rat	Resovist	[38]
		Myocardial infarction & Heart failure	4 mm Quarts Tube	Resovist	[37]
	Eye	Retinal degeneration	S334ter-4 heterozygous transgenic rats	FluidMAG-nanoparticles	[6]
	Liver	No specific condition, Proof of concept	Nude Rats	Ferumoxide PLL complexes	[14]
	Knee	Cartilage injury	*In vitro* model	Positively charged ferric oxide nano-composites	[50]
		Cartilage injury	Mini pig	Ferucarbotran	[51]
Bone marrow stromal cell	Spine	Spinal cord injury	Sprague–dawley rat	poly-L-lysine-coated SPIONs	[52]
Neural progenitor cell	Spine	Spinal cord injury	Sprague–dawley rat	RGDS peptide magnetic bead complex	[53]
Cardiosphere-derived tem cell	Heart	Myocardial infarction	Wistar kyoto rats	Ferumoxytol	[11]
Exogenous bone marrow-derived stem cell (CD45-positive)	Heart	Myocardial infarction	Wistar kyoto rats	Ferumoxytol	[54]
Endogenous stem cell (CD34-positive)	Heart	Myocardial infarction	Wistar kyoto rats	Ferumoxytol	[54]
Endothelial progenitor cell	Vasculature	Common carotid artery injury	Sprague–dawley rat	Feridex	[16]
Bovine aortic endothelial cell	Vasculature	Reendothelialization deficiency after common carotid artery angioplasty	Sprague–dawley rat	Polylactide MNP	[25]
Retinal pigment epithelial cells	Eye	Choroidal neovascularization	*In vitro* model	Magnetite cationic liposomes	[55]

### 4.1. Delivery of Mesenchymal Stem Cells for Spinal Cord Injury

There is currently no available restorative therapy for spinal cord injury in humans. The crippling condition, depending on the severity of the injury, is associated with long-lasting pain and spasticity, and can lead to losses in voluntary movement and sensory abilities, especially tactile [7]. Tukmachev *et al.* used poly-L-lysine SPION-tagged mesenchymal stem cells to treat spinal cord injuries in rats. The rats were injured at lesion site Th10 of the spinal cord and injected intrathecally 10 cm away from the site at the L5-L6 level with 5 × 10^5^ MSCs. Three experimental groups were used: labelled MSCs plus Magnetic Field, non-labelled MSCs plus Magnetic Field, and labelled MSCs with no Magnetic Field. 

The team sought to improve upon the magnetic focusing problems previously reported by using a novel magnetic field approach [52,53]. By creating a “trapping area,” they were able to effectively induce focused cellular migration into a pocket of neutral magnetism, from which they could not later migrate as long as the field was present. They did this by aiming two cylindrical NdFeB magnets, with alike poles facing each other, in the direction of the target site. The efficiency of cellular delivery for the labelled MSC plus Magnetic Field group, defined as the ratio between the “number of cells captured in the lesion site and the total number of cells in the operating range of the spinal cord,” was 45%, compared to just 14% for the non-labelled MSC plus Magnetic Field group and 13% for the labelled MSCs with no Magnetic Field group.

### 4.2. Delivery of Circulating Progenitors for Vascular Injury

Kyrtatos *et al.* delivered progenitor cells at sites of vascular injury using external magnetic devices to help regenerate damage to endothelial layers, which is one of the main initiators of cardiovascular diseases and disorders [16]. They used CD133^+^ endothelial progenitor cells (EPCs) given their involvement in vascular reendothelialization and post-ischemic neovascularization. By targeting EPCs to sites of catheterization in the common carotid artery (CCA) of Sprague-Dawley rats, they hoped to prevent or reduce the incidence of post-angioplasty restenosis, or the re-obstruction of the vessel as a result of scar tissue formation [56]. They used the SPION Endorem (Feridex in the US), activated with neodymium boron permanent disk magnets with a permanent magnetic actuator design that increased the force of the magnetic field at distances farther away from the point of actuation. Thus, they were able to breach the 5mm distance between the skin and the CCA to treat the wound. Results indicate an increase of CD133^+^ cell engraftment to the site of injury by a factor of 5.4 when compared to delivery of the cells without the use of a magnet. A control study evidenced that even when a magnetic field was applied, in the absence of an injury, no cells would permanently target the site, elucidating the interaction between assisted targeting and the need for an underlining biological mechanism to keep the cells engrafted.

### 4.3. Delivery of Mesenchymal Stem Cells for Retinal Degenerations

A host of retinal degenerations affects millions of North Americans, both young and old, and are the leading cause of blindness in developed countries [6]. The most common of these is age-related macular degeneration, but also prevalent are retinitis pigmentosa, and diabetic maculopathy. Yanai *et al.* [6] were able to show that a clinically relevant number of MSCs could be homed into a dystrophic area of the retina without the use of invasive intraocular surgery. More importantly, they were able to administer a significant amount of stem cells intravenously by magnetic targeting at the upper retinal hemisphere, thus adopting the most minimally invasive delivery method available. 

Using Fluid MAG-nanoparticles co-cultured with bone marrow mesenchymal stem cells, this University of Vancouver team created a SPION-labelled MSC. The ferrofluid nanoparticles were composed of a magnetic core protected by a hydrophilic starch coating. They used a retinal degeneration-characterized transgenic rat line that allowed them to model the family of degenerative diseases and treat them. A gold-plated neodymium boron magnetic disk was used to produce the magnetic field at the target site. Two delivery methods were compared, intravitreal, or in the eye injection, and intravenous rat tail injections. Their intravitreal study yielded a 20% coverage of the upper retinal hemisphere and an increase of MSC presence from 9600 to 355,500 cells when compared to studies using no magnetic targeting field in the upper orbit. Their intravenous study suggested a 2.48% coverage of the upper retinal hemisphere and an increase from 4000 to 42,000 cells when compared to studies using no magnetic targeting field in the upper orbit [6]. 

### 4.4. Delivery of Stem Cells for Heart Regeneration

Heart disease remains the number one cause of death in the United States, killing an estimated 2335 people every day, or one person every 37 seconds [11]. Stem cell therapies as a viable avenue for the treatment of heart diseases is a burgeoning, promising field of study. One of the most significant bulwarks in the way of progress in developing actual clinical applications is the lack of substantial long term stem cell engraftment at the target site. In the heart, cell retention rate is only as high as 10% for acute studies (less than 24 hours), and even lower for longer time intervals. The main culprit is identified as the “wash out” factor, or the displacement of the stem cells due to coronary blood flow and heart contraction [11]. 

In a study conducted by Vandergriff *et al.* [11], this wash out was attenuated by the use of FDA-approved Feraheme (ferumoxytol), a SPION used as an anemia drug that was utilized to tag cardiosphere derived stem cells for magnetic targeting. The Feraheme (F), as per a study developed by Frank *et al.* at NIH, was coupled with heparin (H) sulfate and protamine (P) sulfate to form FHP complexes [46]. These complexes were co-incubated with cardiosphere derived stem cells (CDCs) to tag them with the iron nanoparticles. In their study, 500,000 CDCs were intracoronarily injected into female Wistar Kyoto rats as treatment. The rats had previously been subjected to a surgical ischemia/reperfusion procedure that mimicked a heart disease model. The experiment consisted of a control rat group, injected with only PBS, a group treated with cells but subjected to no magnetic field, and a third group treated with cells and subjected to a magnetic field. 

**Figure 2:**
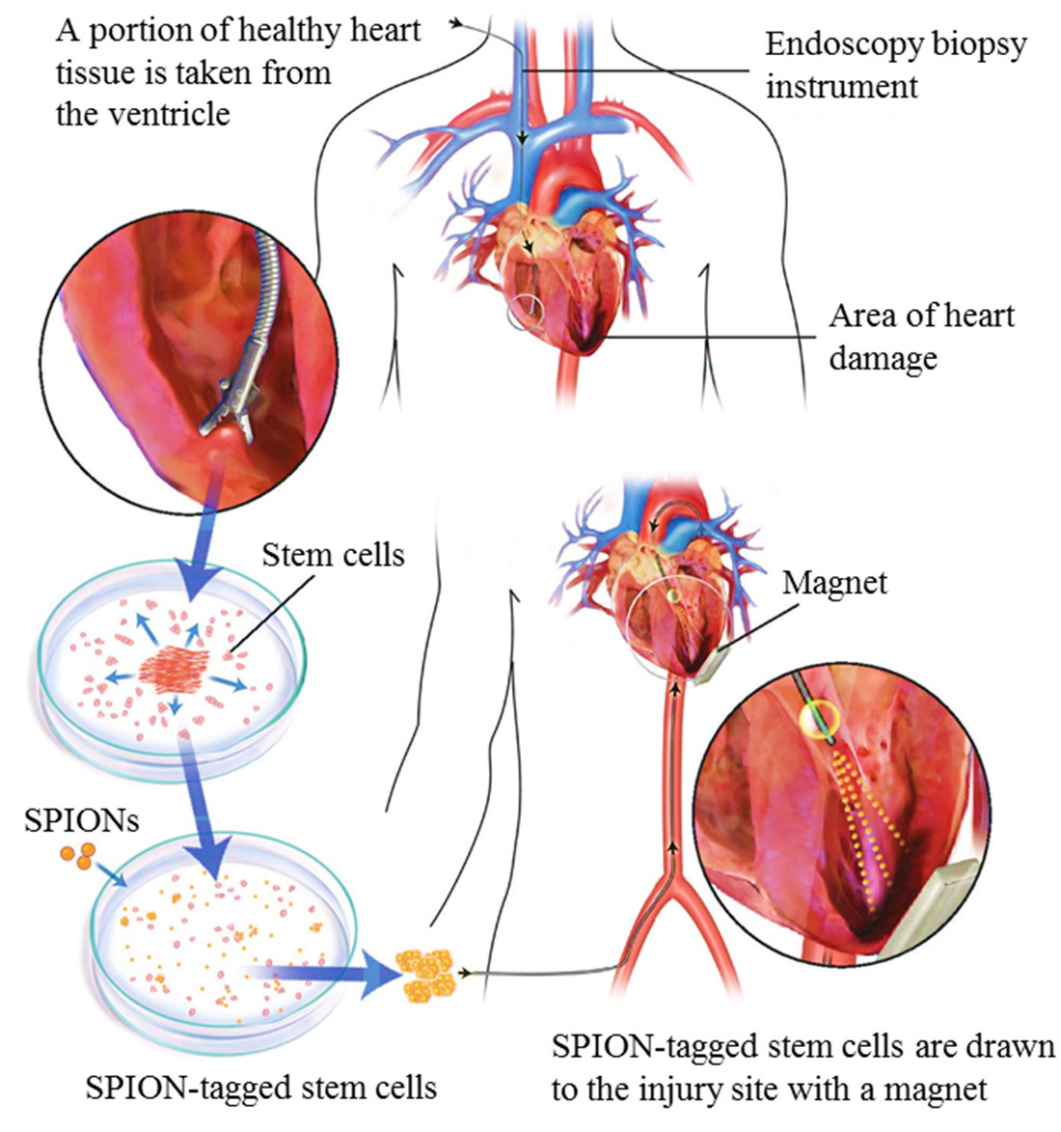
Possible method for acquiring autologous heart stem cells from a human patient, proposed by Cheng *et al.* [11] as part of a model that would use magnetic stem cell targeting for myocardial infarction treatment.

Quantitative PCR revealed that the group treated with the exterior magnetic field exhibited a cell retention rate 3 times greater than the group that was not subjected to the magnetic field. The inflammation marker CD68 was used to compare the level of tissue inflammation between the three groups. All three had similar inflammation characteristics, indicating that neither the magnetic treatment nor the FHP labeling had resulted in increased inflammation. Moreover, the left ventricle remodeling and heart morphology, together with myocardium viability and scar attenuation was altogether better in the group treated with iron-labelled stem cells in the presence of a magnetic field. The application of this technology in humans will require further research, but models with clinical potential are already being proposed, as illustrated in Figure 2.

### 4.5. Delivery of Stem Cells for Cartilage Regeneration

Cartilage repair remains an elusive goal in biomedical engineering. Current treatments yield only limited success and none have been established as a golden standard [50,51]. Studies on cartilage regeneration include bone marrow stimulating techniques, autologous osteochondral grafting, and the use of tissue engineered scaffolds and growth factors [51]. Mesenchymal stem cells have also been intra-articularly injected into defect regions, revealing the need for large numbers of MSCs in order to account for the poor retention of cells in the injection site. Further, as an inadvertent consequence, the use of larger numbers of cells generates loose bodies of scar tissue [51] at the articulation site, affecting its biomechanics. 

In a contribution to recent studies involving the use of MSCs for cartilage repair, Kamei *et al.* [51] developed a novel bulk superconducting magnet system which they used in conjunction with Ferucarbotran (Resovist)-tagged MSCs. Using a mini-pig model, the team created a full thickness cylindrical cartilage defect in the center of the patella of each mini-pig. Each patella was later treated with 5 × 10^6^ cells. Fifteen mini-pigs were divided into three groups: an MSC + Magnetic Field group, an MSC w/out Magnetic Field group, and a control group that was only injected with phosphate-buffered saline. At week 6 and 12 after the surgery, the 15 knees were evaluated by arthroscopic surgery, and at week 12 and 24 histological assessment was conducted.

Results indicated a fast accumulation of magnetically-tagged MSCs after just 10 minutes of external magnetic exposure, and continued retention at the site thereafter. Statistically significant increases in regenerated cartilage were observed in the MSC + Magnetic Field group when compared to the other 2 groups at week 6 and when compared to the phosphate buffered saline group at week 12 using the arthroscopic data. The histological data mirrored the arthroscopic data, with significant increases in hyaline-like cartilage observed in the MSC + Magnetic Field group compared to the fibrous cartilage observe in the other two groups. 

## 5. New Technology: Magnetic Bifunctional Cell Engagers (MagBICE)

The combination of bispecific antibodies with biodegradable iron nanoparticles has recently emerged as a tool for targeted stem cell therapy. The advantage of using bispecific antibodies lies in their dual specificities, which allows them to bind to multiple markers. A recent study by Luma *et al.* used bispecific antibodies to target hematopoietic stem cells to injured myocardium [57]. In this study, the researchers used antibodies with targets to CD45, a hematopoietic marker, and myosin light chain (MLC)-1, a marker for ischemic injury. Thus, the antibody was able to link to the target pathological site with the stem cell intended to provide the curative therapy. 

Analogously, magnetic bifunctional cell engagers (MagBICE) use two types of antibodies conjugated onto iron nanoparticles [54,58]. As in the case of bispecific antibodies, the antibody-nanoparticle conjugate targets the antigen on therapeutic stem cells as well as that on the injured cells, but with the added capacity for magnetic targeting and MRI imaging. Feraheme serves as the iron core of MagBICE. Relative to other iron nanoparticles, Feraheme has the advantage of containing very little free iron, which makes them especially biocompatible, and their carboxylated dextran coating enables a variety of conjugation reactions to attach proteins such as antibodies [58]. The carboxylation of the nanoparticles adds carboxyl groups to the dextran surface that are activated by water soluble 1-ethyl-3-(3-dimethylaminopropyl) carbodiimide (EDAC). The carboxyl groups and EDAC react to create active esters which can bind to the primary amines on the desired antibodies. 

Cheng *et al.* conjugated anti-CD34 (a stem cell marker) and anti-MLC antibodies (an ischemic injury marker) to superparamagnetic nanoparticles [54]. Using a 1.3 Tesla magnetic field, they targeted endogenous CD34^+^ stem cells to the injured myocardium and exerted functional benefits, namely reduced scar mass and size, and preserved infarct wall thickness. They showed that MagBICE combined the elements of molecular targeting, physical enrichment, and noninvasive imaging into one translatable, customizable, and easily synthesized particle.

## 6. Discussion

The magnetically targeted stem cell delivery paradigm is not an isolated advent without competition. Alongside it exist an array of technologies seeking to improve stem cell engraftment post implantation as well. They are as diverse as the field of biomedicine itself, and pull from areas as varied as immunology, genetics, molecular biology, and proteomics. At the leading edge of this wave are antibody, genetic, selectin, and peptide directed targeting approaches [59]. 

Antibodies, with their high affinity to target antigens, can be attached to the surface of cells with the use of palmitated protein-G or A. The two step procedure entails coating the cellular surface with the proteins which intercalate into the phospholipid bilayer of the cells, then incubating the “protein-painted” cells with the desired antibody. Another antibody alternative, briefly introduced in Section 5 of this review, is the use of bispecific antibodies with an affinity to both the pathological region of interest and the cell being delivered. Of the two strategies, the use of protein-G has been the most versatile, while bispecific antibodies prove more difficult to manufacture and have demonstrated poor stability [60].

Genetic manipulation of stem cells by route of transfection can also be a powerful method used to increase cell engraftment. Broadly, genetic-directed cell therapy can be defined as the introduction of DNA or RNA into a cell to induce the expression of cell surface ligands that will aid in its homing and attachment to the target tissue [59]. While surely this is a powerful tool, the regulatory and mechanistic bulwarks involved make its transition into the clinic a hurdle to jump. On the one hand, genetic introduction requires cellular transfection, which can effect cellular viability and itself can be unstable. On the other hand, the combination of gene and cellular delivery in one therapeutic program translates to the potentially push-back from regulatory agencies.

Selectin based approaches are being developed as a way of mimicking the body’s natural immune response to tissue damage or infection; namely, endogenous lymphocyte extravasation [59]. The application of selectin-binding coating on stem cells allows them to penetrate through the endothelial cell and basal membrane layers in order to move from the circulatory system to the site of pathology.

Peptide-directed stem cell engraftment is yet another strategy that can enhance cellular retention. It is a breakthrough technique pioneered at least in part by Kean *et al.* within the last five years that uses phage display to identify targeting peptides with which to coat cells. More so than antibody, gene, or selectin technique, the peptide paradigm has been reported as scalable in manufacture, inexpensive, and capable of producing highly pure products [59].

It can very quickly be ascertained that the potential for pairing engraftment improvement strategies is vast. While the imaging potential of each technique will vary, it is exciting to see how the tools available for cell engraftment studies are manifold and potentially customizable, offering the benefits that come with an assortment of treatment options for patients. The magnetic targeting stem cell delivery protocol suddenly becomes more promising if it can be coupled with any of the mutually beneficial strategies discussed. 

## 7. Conclusion

The use of SPIONs as tools for delivering targeted stem cell therapies to damaged tissues is in its early stages of development, but its potential is promising. However, before the medical research community can transition this technology from benchtop to bedside, large animal studies must be performed, the technology must be scaled up for human applications, and a thorough long term toxicity evaluation of each particle must be performed.

The majority of the animal studies conducted have been on small murine models. In order to develop SPIONs for stem cell therapies on humans a transition into large animal studies will need to take place, as these will more accurately represent human physiological responses and will help address questions involving the scale up of stem cell dosages and magnetic fields used for treatment. While Neodymium Iron Boron magnets are widely applied to animal research, their use in human patients will require a more in depth look at the possible health hazards of strong magnetic fields [61]. 

Safety is paramount in the implementation of magnetic nanoparticle technology for stem cell therapies. While very few *in vitro* studies have reported adverse effects in therapeutic doses, long term *in vivo* studies have not been looked at as extensively [24,30]. To be clinically viable, magnetic nanoparticles must be nontoxic to cells, biodegradable, be compatible with surface modification, must not affect stem cell qualities, must be effective in adequate doses, must not transfer by-products to surroundings, and must be chemically stable in the body [24]. Toxicity risks include the potential for organismal migration, penetration, and accumulation of free iron. The risk for immunological and inflammatory responses in the body is minimized with iron oxide particles when compared to other metallic nanoparticles, but this risk is exacerbated in individuals with genetic dispositions to hemochromatosis, or iron overload [40]. Another important factor to consider is the polymer or other material used to coat the iron oxide core. For example, a toxicity study done on Feridex showed that dextran coatings did not exhibit toxic effects, but HEDP biopolymer coatings had a concentration dependent effect on cell viability [24]. 

In conclusion, magnetic stem cell targeting is at the forefront of studies aimed at improving the efficacy of regenerative medicine. Given its success in guiding stem cells to disease foci, the versatility of SPIONs, and their compatibility with MR imaging modalities, this technology has the potential to expand the horizons of stem cell therapies and solve the problem of low cell retention after stem cell treatment.
